# Interference-assisted kaleidoscopic meta-plexer for arbitrary spin-wavefront manipulation

**DOI:** 10.1038/s41377-018-0113-y

**Published:** 2019-01-09

**Authors:** He-Xiu Xu, Guangwei Hu, Ying Li, Lei Han, Jianlin Zhao, Yunming Sun, Fang Yuan, Guang-Ming Wang, Zhi Hao Jiang, Xiaohui Ling, Tie Jun Cui, Cheng-Wei Qiu

**Affiliations:** 1 Department of Electrical and Computer Engineering, National University of Singapore, Singapore, 117583 Singapore; 2 Air and Missile Defense College, Air force Engineering University, 710051 Xi’an, China; 3 Hunan Provincial Key Laboratory of Intelligent Information Processing and Applications, College of Physics and Electronics Engineering, Hengyang Normal University, 421008 Hengyang, China; 4 School of Natural and Applied Sciences, Northwestern Polytechnical University, 710072 Xi’an, China; 5 Advanced Technique Department, Key Lab of Aeronautics Computing Technique, 710075 Xi’an, China; 6 State Key Laboratory of Millimeter Waves, Southeast University, 210096 Nanjing, China

**Keywords:** Optics and photonics, Optical physics

## Abstract

Achieving simultaneous polarization and wavefront control, especially circular polarization with the auxiliary degree of freedom of light and spin angular momentum, is of fundamental importance in many optical applications. Interferences are typically undesirable in highly integrated photonic circuits and metasurfaces. Here, we propose an interference-assisted metasurface-multiplexer (meta-plexer) that counterintuitively exploits constructive and destructive interferences between hybrid meta-atoms and realizes independent spin-selective wavefront manipulation. Such kaleidoscopic meta-plexers are experimentally demonstrated via two types of single-layer spin-wavefront multiplexers that are composed of spatially rotated anisotropic meta-atoms. One type generates a spin-selective Bessel-beam wavefront for spin-down light and a low scattering cross-section for stealth for spin-up light. The other type demonstrates versatile control of the vortex wavefront, which is also characterized by the orbital angular momentum of light, with frequency-switchable numbers of beams under linearly polarized wave excitation. Our findings offer a distinct interference-assisted concept for realizing advanced multifunctional photonics with arbitrary and independent spin-wavefront features. A variety of applications can be readily anticipated in optical diodes, isolators, and spin-Hall meta-devices without cascading bulky optical elements.

## Fighting interference with interference: Hybrid meta-atoms spin off a kaleidoscope of opportunities

Interferences are typically considered useless and even adverse in highly integrated photonic circuits and metasurfaces. The approach from Cheng-Wei Qiu from the National University of Singapore and colleagues is to exploit interference between hybrid meta-atoms based on pairs of nested microscale rings, each with a small opening. With this counterintuitive recipe, they successfully reported multiplexed functions, i.e., emission of complex vortices and diode-like optical switching devices that manipulates spin lights. By using computer simulations to examine the effects of optical scattering with differently-oriented split rings in periodic arrays, the team identified interference patterns that offered simultaneous control over the helical spin and propagation of circularly polarized light. One proposed metasurface produces narrow beams with pure spin-down states that may have applications in optical tweezing or stealth technology. Another layout generates multiple swirling light vortices by filtering to pure spin-up states. It paves an unexpected paradigm to realizing optical diodes, isolators, and spin-Hall meta-devices without cascading bulky optical elements.

## Introduction

Metasurfaces^1,2^, which are the planar equivalent of metamaterials that are composed of subwavelength meta-atoms, have attracted extensive interest due to their easy fabrication and powerful capabilities in wave manipulation. The flexibility of metasurfaces in controlling the amplitudes and phases of electromagnetic (EM) wave scattering, either individually or simultaneously, has attracted enormous attention from both the science and engineering communities due to their potential applications in modern microwave/optical communication systems^1–14^. Nevertheless, to date, all predefined functions of metasurfaces have been actualized under fixed polarizations, which considerably limit the degree of freedom (DoF) for full-wave controls. This compels us to integrate the features of both phase and polarization to realize precise wavefront multiplexing, which is another crucial DoF of EM waves^15,16^.

The simultaneous manipulation of polarization/spin and phase^17^, which is promising for complicated spin and wavefront multiplexing, is extremely challenging in practice. In the traditional methodology, one must combine linear polarizers, waveplates, and specific phase retarders (prisms or lenses) together. Such a strategy of cascading optical devices leads to bulky systems that exhibit low efficiency and degraded performance. Although chiral metamaterials are considered promising candidates for polarization manipulation^18–21^, the periodic structures in their homogenous profile restrict their capability in shaping the desired wavefronts. Emerging helicity-dependent wavefront control has been realized by Pancharatnam-Berry (PB) phases that are launched by circularly polarized (CP) waves^22–27^. However, the functionalities under left-handed (LCP, spin-up state with a sign of σ−) and right-handed (RCP, spin-down state with a sign of σ + ) CP waves are essentially locked due to the inverse phase profile flipping as the spin state of the incident wave changes. To overcome these limitations, three avenues are available in the open literature: merging the propagation phase and geometric phase across the entire metasurfaces;^28–30^ combining the chirality with the geometric phase;^31,32^ and utilizing the nonflat geometry of the metasurface, which can break the symmetry between LCP and RCP waves^33^. However, these designs either utilized depth-separated two- or multilayered meta-atoms to form a vertical chirality or obtained singular functionality of different modes, which may complicate the design. The study of the physics and criterion for spin-selective functionality is still in its infancy and solutions remain elusive.

**Fig. 1:**
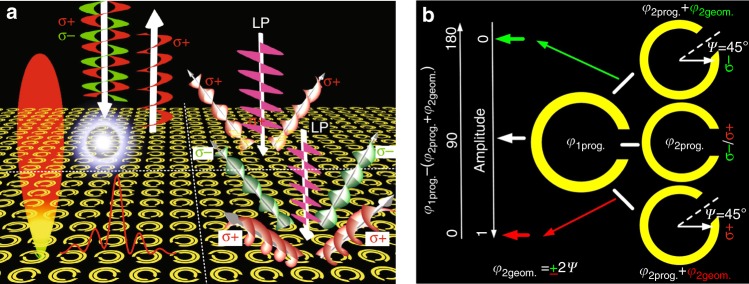
Schematic illustrations of **a** the functionality and **b** interference principle of the proposed kaleidoscopic meta-plexer with arbitrary spin and wavefront controls. Due to the asymmetric CP reflections in each meta-atom, namely, the total absorption and total reflection in the spin-up and spin-down states, the resulting meta-multiplexer realizes the dual functions of low radar cross-section (RCS) for stealth and nondiffractive Bessel-beam wavefront under the CP-wave excitation (left panel of Fig. 1**a**). The spin-selection feature can also be used to flexibly control the number of beams of multiple vortices with a spiral wavefront under the LP wave illumination (right panel of Fig. 1a). The key to engineering constructive and destructive interferences is to seek a meta-atom that is composed of dual parts that have a 90° propagation phase difference and a ± 90° geometric-phase difference at the two spin states, as illustrated in Fig. 1**b**

Here, we propose a novel strategy for enabling a new paradigm for achieving simultaneous spin and wavefront multiplexing (to serve as a kaleidoscopic meta-plexer) by constructive and destructive interferences, as shown in Fig. 1a. The key to this paradigm is to utilize both the propagation and geometric phases to form 180° and 0° phase differences between two counterparts of a meta-atom. This is carried out by flipping the spin states of the CP waves, which distinguishes our work from previous PB designs^22–27^. Therein, diode-like asymmetric CP-wave reflections, with magnitudes of 0 and 1 in the spin-up and spin-down states, can be engineered in each meta-atom (Fig. 1b). To facilitate the out-of-phase and in-phase interferences, a criterion of 90° propagation phase and ± 90° geometric-phase differences is established, which is highly robust for quick and arbitrary designs. For verification, using spatially rotated meta-atoms, two proof-of-concept kaleidoscopic meta-plexers, using spatially rotated meta-atoms, are designed using the feature of asymmetric CP reflections. The first meta-multiplexer exhibits low RCS in the spin-up state and nondiffracting propagation (Bessel wavefront) within the formation zone^34–38^ in the spin-down state. The second meta-multiplexer manifests multiple versatile vortices^30,39–44^ with switched two-to-four pencil beams that carry orbital angular momentum (OAM, twisted phase fronts) at different frequencies under the linearly polarized (LP) wave excitation, thereby demonstrating spin-determined generation and steering of OAM beams. Such distinct spin-selective functionalities are induced by the total absorption of the spin-up wave and the high-efficiency reflection of the spin-down wave. In contrast to previous designs, our approach to realizing the kaleidoscopic functions in one ultracompact metasurface features a monolayer structure, definite physics, and simple design without introducing chirality-selective bulk metamaterials. We firmly believe that, beyond the functions that are demonstrated in our work, additional functionalities are easily realizable by following the proposed design principle.

## Results

### Principle and criterion for the diode-like asymmetric-spin meta-plexer

**Fig. 2:**
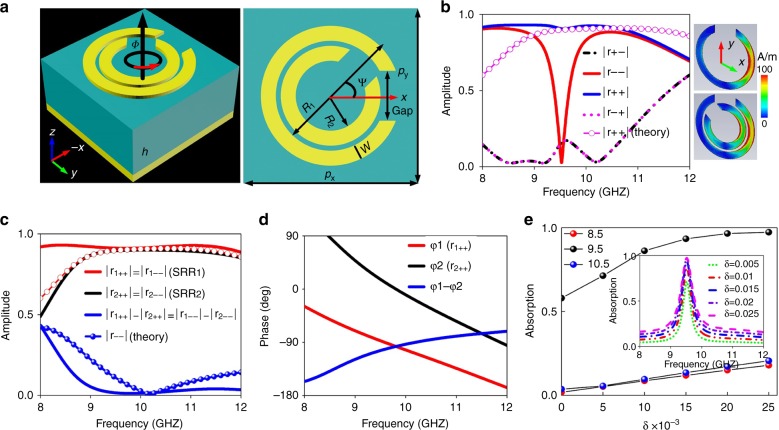
Characterization of the meta-atom with spin-multiplexed asymmetric reflections. **a** An illustration of the topology and structure parameters. **b**, **c** A comparison of FDTD-simulated and theoretically calculated |*r*_++_| and |*r*−−| of the meta-atom under normally incident spin-up and spin-down plane waves. The inset in Fig. 2**b** shows the current distributions (*J*_x_) of both SRR_1_ and composite SRR, illuminated by a plane CP wave. Here, theoretical calculations are based on the interference principle that is discussed above. FDTD-simulated reflective **c** amplitude and **d** phase spectra of the SRR_1_ and SRR_2_ meta-atom. **e** Absorption spectra that are calculated via $a_L =1-r^{2}_{LL} - r^{2}_{RL}$ for various values of *δ*. The geometrical parameters are *R*_1_ = 2.2, *R*_2_ = 1.4, *w* = 0.6, *p*_*x*_ = *p*_*y*_ = 7 (unit: mm), and *ψ* = 45°, and the gap widths of the inner and outer rings are 1.5 and 2 mm, respectively

To realize the new-concept kaleidoscopic CP wavefront control, we first state the interference principle for asymmetric CP reflections and establish a general criterion for design based on a novel meta-atom. The basic building block that is proposed here is a metal-insulator-metal reflection structure on a continuous metallic background (Fig. 2a). The top metal structure is comprised of two split ring resonators (SRRs) that exhibit a local mutual twist of *ψ* = 45°. For convenience, the external and internal SRRs are denoted as SRR_1_ and SRR_2_, respectively. An F4B dielectric board with dielectric constant *ε*_r_ = 4.5, thickness *h* = 3 mm, and loss tangent *δ* = 0.025 is placed between the composite metallic pattern and backed metal ground. Here, two types of phases are involved: the propagation phase, which is induced by parametric variations, and the geometric phase, which is induced by orientation rotations about its axis. By individually controlling the geometrical parameters of SRR_1_ and SRR_2_, an arbitrary propagation (reflection) phase difference, which is expressed as Δ*ϕ* = *ϕ*_*1*_–*ϕ*_*2*_, can be engineered between two reflections of *r*_1++_ and *r*_2++_, or those of *r*_1−−_ and *r*_2−−_ under spin-down and spin-up wave stimulations. Such a propagation phase difference has no role in controlling the wavefront but induces helicity selectivity. Here, *r*_1++_ and *r*_2++_ (*r*_1−−_ and *r*_2−−_) denote the copolarization reflections of SRR_1_ and SRR_2_ in the spin-down (spin-up) state, respectively.

Initially, we consider a CP wave with normal incidence on the meta-atom of SRR_1_ or SRR_2_ with a common global rotation angle *Φ*. The reflections will gain an additional geometric phase of 2*Φ* according to PB phase theory. Similar to the V-shaped antenna^1^, SRR_1_ and SRR_2_ exhibit symmetric and asymmetric modes parallel and perpendicular to the symmetric axis under plane CP-wave excitation. This is because any CP wave can be considered composed of two equally contributing orthogonal LP components. At such resonant modes, several reflection zeros are manifested on the *r*_−+_ and *r*_+−_ spectra. By cascading these resonant modes of the two SRRs, i.e., merging them into a composite element, broadband high-efficiency reflections can be engineered, which can be derived from the Jones matrix of *r*_1_ and *r*_2_ (see Fig. S1a and additional details in Supplementary Materials). In the case of negligible weak coupling between SRR_1_ and SRR_2_, the reflections of the composite meta-atom can be simplified as

1 $$\begin{array}{l}E_{{\mathrm{com + }}} \propto \left| {r_{1 + + }} \right|E_i{\mathrm{e}}^{{\it{i}}\left( {\varphi _1 + 2\Phi } \right)} + \left| {r_{2 + + }} \right|E_i{\mathrm{e}}^{{\it{i}}\left( {\varphi _2 + 2\Phi \pm \psi } \right)}\\ E_{{\mathrm{com - }}} \propto \left| {r_{1 - - }} \right|E_i{\mathrm{e}}^{{\it{i}}\left( {\varphi _1 - 2\Phi } \right)} + \left| {r_{2 - - }} \right|E_i{\mathrm{e}}^{{\it{i}}\left( {\varphi _2 - 2\Phi \mp \psi } \right)}\end{array}$$

In a full reflection scheme, it is easy to show that |*r*_1++_| ≈ |*r*_2++_| ≈ 1 and |*r*_1−−_| ≈ |*r*_2−−_| ≈ 1. Then, the residuals *ϕ*_*1*_, *ϕ*_*2*_, *Φ*, and *ψ* play a determinant role in obtaining CP asymmetric reflections. To maximize *E*_com−_ or *E*_com+_ while minimize the other, the following criteria should be satisfied:

2 $$\begin{array}{l}\varphi _1 - \varphi _2 \mp 2\psi = 0\\ \hskip 10pt \varphi _1 - \varphi _2 \pm 2\psi = 180\end{array}$$

From Eq. (2), we immediately obtain the exclusive solution of Δ*ϕ* = −90° and *ψ* = 45° under the spin-down wave; in contrast, Δ*ϕ* = −90° and *ψ* = −45° under the spin-up wave, which are general criteria for diode-like spin controls. The abovementioned phase requirement is only related to the local orientations and dimensions of the two-SRR meta-atom. Using the above criteria, we can realize full reflection of the spin-down/up wave through the in-phase constructive interference and total suppression of the spin-up/down wave through the out-of-phase destructive interference in the spin-down/up state. According to Eq. (1) and (2), the amplitude of all reflections in the Jones matrix can be tuned by cautiously choosing an appropriate value of *ψ*. This prediction finds strong support from Supplementary Fig. S1b, where |*r*_−−_| and |*r*_++_| can be continuously and individually modulated by controlling the local *ψ* within −180^o^–0° and 0^o^–180°. Moreover, the phase can be independently and continuously tuned by the global *Φ* without affecting the amplitude. Although the above criteria, which are derived from interference, exclude the consideration of mode coupling between two SRRs, our coupled mode analysis (Supplementary Fig. S1c) confirms that the coupling is very weak. Therefore, our theory provides an intuitive and nontrivial guideline for asymmetric-spin controls. Moreover, such weak coupling can be directly observed in the inset of Fig. 2b, where the current density and distributions (*J*_x_) are almost the same on an individual SRR_1_ and a composite SRR.

### Design and verification of the diode-like asymmetric-spin meta-plexer

Based on the established criteria, it is easy to design a spin-selective meta-plexer that operates at an arbitrary frequency. First, we roughly determine the structures of SRR_1_ and SRR_2_ via individual design of the propagation phase. Then, we finalize the meta-atom layout via finely optimizing the parameters of the hybrid SRR_1_ and SRR_2_ with the required orientations. For verification, we characterize the proposed meta-atom that is designed from the above criteria via finite-difference time-domain (FDTD) simulations. Figure 2b shows that the handedness of the reflected beam is preserved for both spin-up and spin-down waves, while the cross-polarization reflections are maintained extremely small across a broadband. Moreover, |*r*_++_| remains above 0.71 over the entire observation band, while |*r*_−−_| undergoes a sudden drop, with its magnitude approaching null at 9.5 GHz, thereby enabling a maximum extinction ratio of |*r*_++_|/|*r*_−−_| = 33.3. Such diode-like asymmetric reflections indicate a giant circular dichroism. The FDTD and theoretically calculated reflection spectra are in reasonable agreement, thereby verifying the physical mechanism of destructive and constructive interferences, as illustrated in Fig. 2b, c, in which the curve that is composed of circle symbols is the theoretical result. The proposed approach is strongly supported by Fig. 2c, d, where the calculated values of |*r*_1++_| and |*r*_1−−_| are almost the same as those of |*r*_2++_| and |*r*_2−−_|, while the phase difference Δ*ϕ* = *ϕ*_*1*_-*ϕ*_*2*_ between them is nearly −90°. These results correspond exactly to the criteria that are specified by Eq. (2). The slight deviation between theory and simulations, especially for the narrower-band absorption in the latter case, is attributed to the slightly altered reflection amplitude of SRR_2_ under the orientations of *ψ* = 0° and 45^o^ and weak coupling between two closely spaced SRRs. The slight deviation of the reflections between theory and simulations at off-interference (off-*f*_0_) frequencies and narrower-band absorption in the latter case is attributed to the inevitable coupling between SRR_1_ and SRR_2_ when the frequency exceeds *f*_0_. This is because in the theoretical prediction of the composite meta-atom, the reflection responses of two individual SRR_1_ and SRR_2_ meta-atoms were modeled separately to yield guidelines regarding constructive and destructive interferences. However, in full-wave simulations, an integrated meta-atom that involved two SRRs (one SRR in the vicinity of the other) was characterized. Nevertheless, this has little effect on identifying the interference mechanism by which perfect coherence is clearly observed near the center frequency. The almost constant reflection amplitudes of *r*_++_, *r*_−−_, *r*_+−_, and *r*_−+_ with *Φ* and the ideal PB phase of *r*_++_ with 2*Φ* can be clearly observed in Supplementary Fig. S1d-S1f. Such robust amplitude and phase profiles against *Φ* are crucial for high-efficiency wavefront control.

The spin-selective asymmetric reflection is not induced by the loss of the dielectric board. This is supported by Fig. 2e, where high absorptions are sustained even if the loss tangent, which is denoted as *δ*, reaches zero. Nevertheless, the absorption rate of *r*_++_ increases slightly with δ in the on- and off-interference regions and more than 95% reflection efficiency is expected when widely available dielectrics with *δ* = 0.005 are adopted. The near-perfect absorption in the spin-up state originates from the strong localized fields (dissipative loss) that are induced by mutual interference between SRR_1_ and SRR_2_ (inset of Supplementary Fig. S1d).

**Fig. 3:**
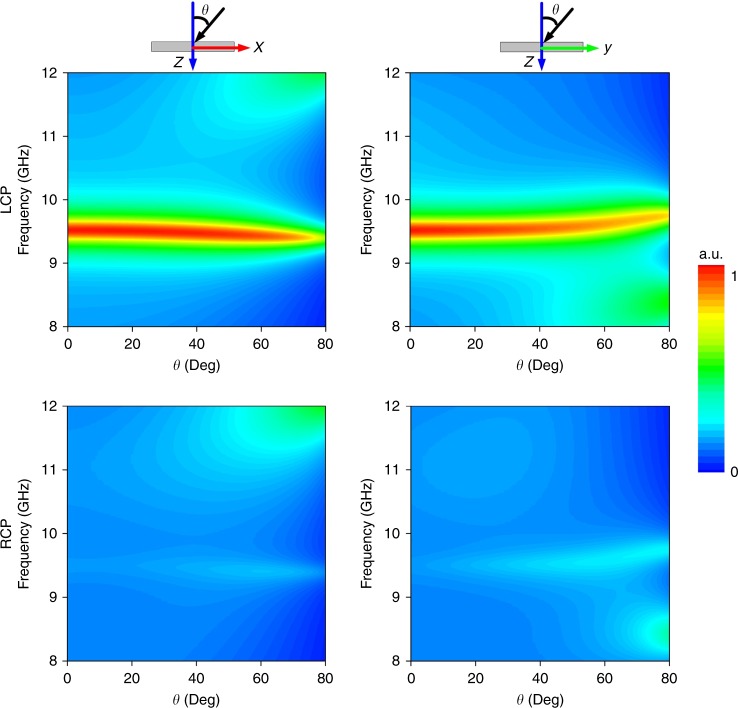
Angle-immune performance of the diode-like asymmetric-spin multiplexer. Here, the FDTD-simulated absorption spectra of $a_L$ (top row) and $a_R =1-r^{2}_{RR} - r^{2}_{LR}$ (bottom row) in the spin-up and spin-down states are presented as functions of the incidence angle *θ* and frequency when EM waves are incident on the *xz*-plane (left panel) and *yz*-plane (right panel), respectively

The proposed meta-multiplexer for asymmetric-spin control performs robustly at nearly full-angle incidences, as depicted in Fig. 3. Again, a remarkable diode-like asymmetric absorption is clearly observed from *a*_L_ and *a*_R_ in the spin-up and spin-down states on both the *xz* and *yz* incident planes. Moreover, the near-unity and null asymmetric absorptions in the spin-up and spin-down channels are preserved over a wide range of incidences. Even when the incident angle reaches 80°, the absorption of *a*_L_ exceeds 68% in the *xz*-plane and 76% in the *yz*-plane. The absorptions in the two states maintain a large contrast ( > 50%), even under an incident angle of up to 65°, thereby indicating a robust wide-angle absorption behavior. The slight deviation of the absorption behavior *a*_L_, namely, red and blue frequency shifts on the *xz*- and *yz*-planes at large incident angles, is attributable to the simultaneous breaking of mirror and rotational symmetry. Such high-contrast incidence-insensitive diode-like reflections are highly beneficial for applications in single-mode devices of CP lights.

### Multiplexed Bessel beam and RCS reduction

**Fig. 4:**
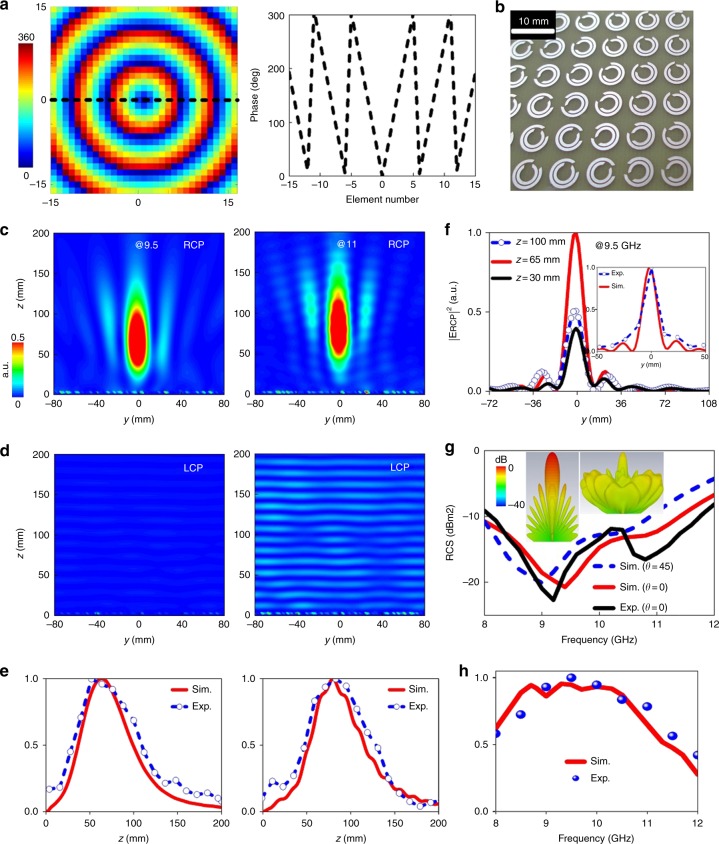
Characterization of the first meta-multiplexer with Bessel-beam generation and RCS reduction. **a** Phase distribution across the axicon aperture and central line. **b** A magnified view of the fabricated prototype. FDTD-simulated *E*-field intensity distributions at 9.5 and 11 GHz under **c** spin-down and **d** spin-up plane-wave illuminations. The near-field *E*_RCP_ intensities in the spin-down state **e** along the propagating direction (*z*-axis) at *x* = 0 and **f** on various *yz*-planes with *z* varying from *z* = 30 to *z* = 125 mm away from the meta-multiplexer. **g** The normalized far-field RCS reduction spectrum to the perfect electric conductor (PEC) ground with the same size and **h** near-field *E*_RCP_ intensity spectra at the focal point (maximum intensity) under the illumination of spin-up and spin-down waves. The inset of Fig. 4**g** shows the 3D scattering patterns of the meta-plexer and PEC with the same size at 9.5 GHz. Here, all fields are normalized to its maximum

In the following, we demonstrate that our strategy can engineer multiplexed distinctive wavefront controls using such CP asymmetric reflections. The first kaleidoscopic meta-plexer is designed to generate nondiffractive Bessel beams^34–38^. The meta-multiplexer targeting at 10 GHz is composed of 31*31 pixels and occupies an area of 217*217 mm^2^. To form the diffraction-free focusing wavefront in the spin-down state, the imparted phase should satisfy^34^

3 $$\varphi _{{\rm{Axicon}}} = \sin \left( {\tan ^{ - 1}\left( {\frac{R}{F}} \right)} \right) \ast \frac{{2\pi }}{\lambda } \ast \sqrt {x^2 + y^2}$$

in which *F* = 100 mm is the long depth of focus and *R* = 108.5 mm is half the axicon aperture, as shown in the 2D hyperbolic phase profile and liner phase profile along *y* = 0 (dashed) in Fig. 4a. Then, the meta-multiplexer layout can be easily mapped out by spatially varying the global orientations of meta-atoms with constant geometrical parameters, as illustrated in Supplementary Fig. S2a and Fig. 4b. For experimental verifications, a proof-of-concept sample is fabricated via the printed circuit board technique. The spin-selective nondiffracting reflection and broad-angle RCS reduction are measured in a microwave anechoic chamber (see Supplementary Fig. S4 for the experimental details).

Figure 4c, d present the color maps of the *E*-field intensity distributions on the *yz*-plane at 9.5 and 11 GHz for intuitionistic study. Distinct behaviors are clearly observed under two opposite-helicity channels. For the spin-down channel, we observe a nondiffracting propagation (Bessel beams) behavior along the *z*-axis across a desirable distance over a wide range of frequencies, which serves as the needle beam. In sharp contrast, weak and dispersed energy distributions with diverged fields are observed for the spin-up channel. Moreover, the near fields even approach null across the entire plane at 9.5 GHz, where the extinction ratio, which is calculated as the scale of the maximum power of the two channels, is evaluated as more than 50. Such a desirable distinct function is attributable to the diode-like asymmetric reflections of the utilized meta-atoms. Figure 4e, f quantitatively compare the FDTD-simulated and experimentally measured *E*-field intensities across the propagating direction (*x* = 0) and three *yz*-planes, respectively. A reasonable agreement of results is observed between simulations and measurements. As expected, the energy is dominantly confined close to the optical axis and extends along the axis for an appreciable distance without diffraction (~75 mm by 3 dB energy attenuation). The half-power beamwidth in the transverse plane is ~15 mm and remains almost constant as the frequency varies, as shown in Fig. 4f, where satisfactory Bessel-like profiles of electric fields with the first zero are clearly observed at 9.5 GHz. Results at other off-interference frequencies are presented in Supplementary Figs. S2c-S2d. As expected, in Fig. 4g, the backward RCS has been significantly reduced by more than 7 dB across 8–12 GHz, especially near 9.5 GHz, where the RCS reduction reaches −20.7 dB, as clearly illustrated in the 3D scattering patterns at 9.5 GHz in the inset. Most importantly, the low-RCS behavior is robust against the incidence changes and the RCS reduction behavior is preserved to better than −6 dB, even up to *θ* = 45°.

Finally, we show the E-field intensity spectra in Fig. 4h at the focal point and use them to quantitatively evaluate the working bandwidth. As expected, the E-field intensity reaches its maximum at ~*f*_0_ = 10 GHz, thereby demonstrating the highest capability of Bessel-beam generation. However, the capability deteriorates when the frequencies exceed *f*_0_ since the required dispersive hyperbolic phase profile is no longer strictly fulfilled. The working bandwidth, which is characterized by half-power decay, is ~3.4 GHz (8–11.4 GHz), which is equivalent to a fractional bandwidth of 34%. Slight deviations between the numerical and experimental results are likely induced by the inaccurate alignment of the meta-multiplexer and the feed horn and the imperfect nonplanar incoming wavefront. In addition to the generation of a Bessel beam by the impinged wavefront, other functions (e.g., focusing and twisted wavefronts) are readily realized by altering the local rotations of the proposed meta-atoms, which can be highly useful in various functional devices.

### Multiplexed vortices with versatile beams

**Fig. 5:**
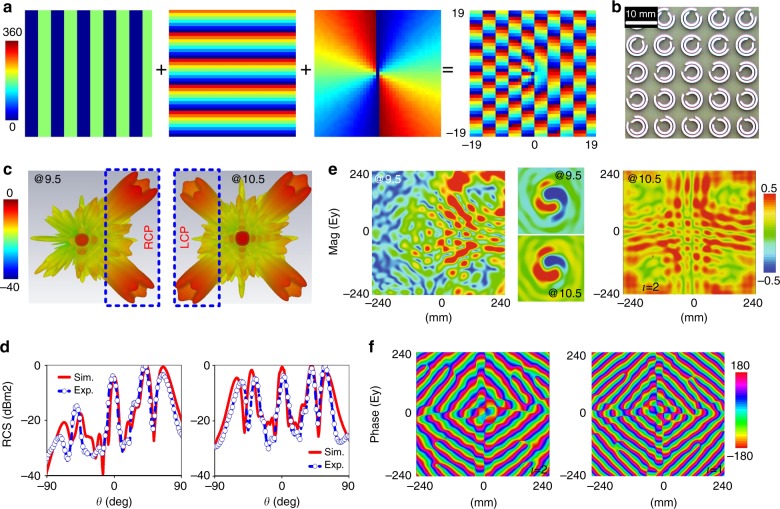
Characterization of the second meta-multiplexer with versatile vortices via illuminating the plate with spin-up and spin-down plane waves. **a** Illustration of how to obtain the composite phase profiles via merging three types of phases. **b** A magnified view of the fabricated sample. Normalized FDTD-calculated and experimentally measured far-field **c** 3D and **d** 2D scattering patterns along *φ* = 45° at 9.5 and 10.5 GHz. **e** Measured near-field *E*_y_ distributions at 9.5 and 10.5 GHz, including Mag(*E*_y_) at *z* = 250 mm (8.75 *λ*) above the meta-multiplexer (left and right panels) and Real(*E*_y_) information on a cross-section that is vertical to one Bessel beam (middle inset). **f** The FDTD-simulated Phase(*E*_y_) at 10.5 GHz for a meta-multiplexer that is carrying OAM modes of *l* = 1 and *l* = 2. The element phase in each supercell has 0, 0, 0, 0, π, π, π, and π along the *x*-direction and 0, π/4, π/2, 3π/4, π, 5π/4, 3π/2, and 7π/4 along the *y*-direction

Here, we further demonstrate that the CP asymmetric reflections can be utilized to flexibly manipulate multiplexed pencil beams that carry vortex information, or OAM, in two opposite-helicity channels. By encoding several phase profiles into a single metasurface plate^45^, a composite wavefront and versatile functionalities can be realized, which is promising for capacity enlargement. Here, three phase profiles are mixed, as shown in Fig. 5a: a spiral phase, namely, *ϕ*_3_(*x*, *y*) = exp(−i*lϕ*), where *l* = 1 and 2 denoted the topological charges of vortices, and two linear gradient phases, namely, *ϕ*_1_(*x*, *y*) = *ξ*_*x*_*x* and *ϕ*_2_(*x*, *y*) = *ξ*_*y*_*y*, along the *x*-and *y*-directions with *ξ*_*x*_ = *ξ*_*y*_ = 0.54*k*_0_ and eight meta-atoms in each supercell. The meta-multiplexer contains 39*39 meta-atoms and occupies a total area of 273*273 mm^2^, as illustrated in the chip-level and pixel-level layouts that are shown in Fig. 5b and Supplementary Fig. S3. The main physics of generating multiple vortices with versatile numbers of beams can be understood as follows: First, a pair of symmetric spin-down beams with OAM undergo opposite reflections along the + /−*x*-directions with an elevation angle of *θ*_1_ = sin^−1^(*ξ*_*x*_/*k*_0_), while a single spin-down beam of OAM is shaped along the + *y*-direction with *θ*_2_ = sin^−1^(*ξ*_*y*_/*k*_0_) in the spin-down state. Then, two symmetric beams interfere with the single beam and eventually form two spin-down beams in the direction of $\theta {\mathrm{ = }}\sin ^{ - 1}\left( {\sqrt {\sin ^2\theta _1 + \sin ^2\theta _2} } \right)$ and *ϕ* = 90° ± tan^−1^(sin*θ*_1_/sin*θ*_2_), where *ϕ* is the azimuth angle. In a similar manner, two symmetric spin-up beams are directed with the same angle along the + /−*x*-axis, while a single spin-up beam is formed along the –*y*-direction in the spin-up state. Again, these beams form two interfering spin-up beams at $\theta {\mathrm{ = }}\sin ^{ - 1}\left( {\sqrt {\sin ^2\theta _1 + \sin ^2\theta _2} } \right)$ and *ϕ* = 270° ± tan^−1^(sin*θ*_1_/sin*θ*_2_). Finally, four vortices with different helicities (two spin-ups and two spin-downs) are simultaneously engineered for our meta-multiplexer under a plane LP wave with normal incidence (a combination of spin-up and spin-down waves). The key to manipulating the number of vortex beams is to control the spin-up reflections based on our previously established criteria.

Figure 5c illustrates the 3D scattering patterns in the far field at 9.5 and 10.5 GHz. As expected, four symmetric vortices with null amplitude in the centers are precisely directed toward the spatial angles that are predicted theoretically at 10.5 GHz. However, the number of pencil vortices decreases to two at 9.5 GHz, with two spin-up beams being totally absorbed. The generation of two asymmetric spin-down vortices can be observed within 9.2~9.7 GHz. The performance can be further evaluated quantitatively in Fig. 5d, where the simulated and measured 2D patterns along *ϕ* = 45° agree well, thereby indicating one and two beams each with central singularities at 9.5 GHz and 10.5 GHz. The extinction ratio of spin-down to spin-up vortex beams is measured as 18 dB. The slightly raised normal reflections are attributable to the interferences that are induced among several phase profiles. To further evaluate the OAM vortex wavefronts, Fig. 5e shows the *E*-field (*E*_y_) distributions at 10.5 GHz and *z* = 250 mm above the meta-multiplexer with the cross-section perpendicular to one vortex beam. As expected, the doughnut-shaped intensity distribution is almost uniformly formed in four regions at 10.5 GHz. However, at 9.5 GHz, only two regions are filled with strong doughnut-shaped intensity, while the intensity in the left half-region is weak. Most importantly, the pure spiral phase fronts with two arms (*l* = 2) can be clearly observed from the perpendicular cross-section. The wavefront above the meta-multiplexer is a combination of anomalous deflections along *ϕ* = 45° and 135° and a helical wavefront, as presented in the quad fingerprint line-spiral wavefronts in Fig. 5f. The one and two forks (optical singularity) in each region correspond to vortex beams that are carrying OAM modes of *l* = 1 and *l* = 2. Additional scattering patterns at other off-interference frequencies and a spiral phase wavefront with doughnut-shaped intensity are plotted in Supplementary Figs. S3b-S3e.

## Discussion

In summary, we have proposed a new strategy for CP asymmetric diode-like reflections that is based on constructive and destructive interferences. We established criteria that provide a general guideline for engineering an arbitrary extinction ratio of the CP waves using arbitrary structures. For verification, a type of ultrathin planar meta-atom is proposed and demonstrated to selectively reflect the spin-down waves with a high efficiency of 95% while completely absorbing the spin-up waves. To demonstrate its promising applicability, a kaleidoscopic meta-plexer that merges diffraction-free Bessel-beam generation and wide-angle low-RCS for stealth in one plate and a kaleidoscopic meta-plexer for vortex-beam generation with versatile beam numbers are numerically and experimentally characterized. Desirable kaleidoscopic manipulations of spin and wavefronts have been verified. Moreover, the interference-assisted paradigm can be readily adopted in the high-frequency range (visible or infrared)^46^ if any two individual resonators are weakly coupled and in a transmissive scheme that provides a quasi-uniform transmission rate; see Supplementary Fig. S4 and Fig. S5. Our strategy opens an avenue to flexibly controlling both the helicity and wavefront of CP lights with unprecedented ability by integrating artificial intelligence for purposive purposeful and smart selections.

## Materials and method

### Numerical characterizations

All numerical designs and characterizations are performed via FDTD simulations. In calculations of the reflective amplitudes/phases of the metasurface, we only studied a meta-atom with periodic boundary conditions assigned at its four bounds and a Floquet port that is placed 20 mm away from the meta-atom plane. In near-field and far-field calculations, a square-shaped meta-plexer that is formed by a set of spatially rotated meta-atoms is investigated with open boundary conditions applied at its four bounds. In all scenarios, the meta-plexers/meta-atoms are illuminated by a normally incident plane wave that is in the LP, spin-up or spin-down state.

### Microwave experiments

To avoid interference from the environment, all far-field (FF) and near-field (NF) microwave experiments are performed in an anechoic chamber. Supplementary Fig. S6 shows the experimental setup. In NF measurements, the meta-multiplexer sample was excited by a CP horn with an axial ratio of less than 3.5 dB and a voltage-standing-wave ratio of less than 2.5 at 6–18 GHz. They were fixed with a distance of 1 m. A 15-mm-long monopole antenna, which functioned as the receiver, was placed between the sample and the CP horn and was linked to an N5230C Agilent vector network analyzer to record the static EM signals. The monopole was fixed to a 2D electronic step motor that can move automatically in a maximum area of 0.6 m × 0.6 m with a step resolution of 3 mm. By shifting the monopole orientation along the *x*- and *y*-directions, both the local *E*_*x*_ and *E*_*y*_ fields can be obtained (with both amplitude and phase). Then, the spin-up and spin-down components can be calculated as $E_{{\rm{LCP}}} = \frac{1}{{\sqrt 2 }}\left( {E_x - iE_y} \right)$ and $E_{{\rm{LCP}}} = \frac{1}{{\sqrt 2 }}\left( {E_x + iE_y} \right)$ by incorporating the measured information. By altering the relative position of the meta-multiplexer and 2D monitor, we can obtain the field information in the *xz*-, *yz*-, and *xy*- planes. In all simulated and measured near-field maps, the incident signal in free space was subtracted from the total fields. In the FF RCS measurements, two CP horns are adopted as the transmitter and receiver and are displaced 1 m from the sample. The receiving CP horn, which was aligned with the meta-multiplexer, rotated freely to record the signal that was scattered within −90^o^ < *θ*_r_ < 90°. In the FF quad-beam pattern measurements, the meta-multiplexer was fed by a small LP conical horn at a focal point of *F* = 100 mm and both of them were secured on a large rigid foam that is capable of rotating freely along the foam’s axial center. The CP receiver was placed 10 m away to record the far-field signals.

## Supplementary information

Interference-Assisted Kaleidoscopic Meta-plexer for Arbitrary Spin-Wavefront Manipulation

## References

[1] Yu NF (2011). Light propagation with phase discontinuities: generalized laws of reflection and refraction. Science.

[2] Sun SL (2012). Gradient-index meta-surfaces as a bridge linking propagating waves and surface waves. Nat. Mater..

[3] Liu LX (2014). Broadband metasurfaces with simultaneous control of phase and amplitude. Adv. Mater..

[4] Kim M, Wong AMH, Eleftheriades GV (2014). Optical Huygens’ metasurfaces with independent control of the magnitude and phase of the local reflection coefficients. Phys. Rev. X.

[5] Wang Q (2016). Broadband metasurface holograms: toward complete phase and amplitude engineering. Sci. Rep..

[6] Lee GY (2018). Complete amplitude and phase control of light using broadband holographic metasurfaces. Nanoscale.

[7] Ding J, An SS, Zheng B, Zhang HL (2017). Multiwavelength metasurfaces based on single-layer dual-wavelength meta-atoms: toward complete phase and amplitude modulations at two wavelengths. Adv. Opt. Mater..

[8] Cui TJ, Qi MQ, Wan X, Zhao J, Cheng Q (2014). Coding metamaterials, digital metamaterials and programmable metamaterials. Light Sci. Appl..

[9] Aieta F, Kats MA, Genevet P, Capasso F (2015). Multiwavelength achromatic metasurfaces by dispersive phase compensation. Science.

[10] Xu HX (2018). Wavenumber-splitting metasurfaces achieve multi-channel diffusive invisibility. Adv. Opt. Mater..

[11] Zhu BO (2014). Dynamic control of electromagnetic wave propagation with the equivalent principle inspired tunable metasurface. Sci. Rep..

[12] Chen K (2017). A reconfigurable active Huygens’ metalens. Adv. Mater..

[13] Xu HX (2016). Dynamical control on helicity of electromagnetic waves by tunable metasurfaces. Sci. Rep..

[14] Qin F (2016). Hybrid bilayer plasmonic metasurface efficiently manipulates visible light. Sci. Adv..

[15] Gansel JK (2009). Gold helix photonic metamaterial as broadband circular polarizer. Science.

[16] Hao JM (2007). Manipulating electromagnetic wave polarizations by anisotropic metamaterials. Phys. Rev. Lett..

[17] Li JX (2015). Simultaneous control of light polarization and phase distributions using plasmonic metasurfaces. Adv. Funct. Mater..

[18] Pfeiffer C, Zhang C, Ray V, Guo LJ, Grbic A (2014). High performance bianisotropic metasurfaces: asymmetric transmission of light. Phys. Rev. Lett..

[19] Menzel C (2010). Asymmetric transmission of linearly polarized light at optical metamaterials. Phys. Rev. Lett..

[20] Mutlu M, Akosman AE, Serebryannikov AE, Ozbay E (2012). Diodelike asymmetric transmission of linearly polarized waves using magnetoelectric coupling and electromagnetic wave tunneling. Phys. Rev. Lett..

[21] Xu HX, Wang GM, Qi MQ, Cai T, Cui TJ (2013). Compact dual-band circular polarizer using twisted Hilbert-shaped chiral metamaterial. Opt. Express.

[22] Zhou JX (2018). Broadband photonic spin hall meta-lens. ACS Nano.

[23] Chen XZ (2012). Dual-polarity plasmonic metalens for visible light. Nat. Commun..

[24] Zheng GX (2015). Metasurface holograms reaching 80% efficiency. Nat. Nanotechnol..

[25] Zhang L, Liu S, Li LL, Cui TJ (2017). Spin-controlled multiple pencil beams and vortex beams with different polarizations generated by Pancharatnam-Berry coding metasurfaces. ACS Appl. Mater. Interfaces.

[26] Xu HX (2018). Deterministic approach to achieve broadband polarization-independent diffusive scatterings based on metasurfaces. ACS Photonics.

[27] Wen DD (2015). Helicity multiplexed broadband metasurface holograms. Nat. Commun..

[28] Arbabi A, Horie Y, Bagheri MM, Faraon A (2015). Dielectric metasurfaces for complete control of phase and polarization with subwavelength spatial resolution and high transmission. Nat. Nanotechnol..

[29] Mueller JPB, Rubin NA, Devlin RC, Groever B, Capasso F (2017). Metasurface polarization optics: independent phase control of arbitrary orthogonal states of polarization. Phys. Rev. Lett..

[30] Devlin RC, Ambrosio A, Rubin NA, Mueller JPB, Capasso F (2017). Arbitrary spin-to–orbital angular momentum conversion of light. Science.

[31] Wang ZJ (2016). Circular dichroism metamirrors with near-perfect extinction. ACS Photonics.

[32] Jing LQ (2017). Gradient chiral metamirrors for spin-selective anomalous reflection. Laser Photonics Rev..

[33] Burch J, Di Falco A (2018). Surface topology specific metasurface holograms. ACS Photonics.

[34] Durnin J, Miceli JJ, Eberly JH (1987). Diffraction-free beams. Phy Rev. Lett..

[35] Qi MQ, Tang WX, Cui TJ (2015). A broadband Bessel beam launcher using metamaterial lens. Sci. Rep..

[36] Pfeiffer C, Grbic A (2014). Controlling vector Bessel beams with metasurfaces. Phys. Rev. Appl..

[37] Aieta F (2012). Aberration-free ultrathin flat lenses and axicons at telecom wavelengths based on plasmonic metasurfaces. Nano. Lett..

[38] Chen WT (2017). Generation of wavelength-independent subwavelength Bessel beams using metasurfaces. Light Sci. Appl..

[39] Heckenberg NR, McDuff R, Smith CP, White AG (1992). Generation of optical phase singularities by computer-generated holograms. Opt. Lett..

[40] Thidé B (2007). Utilization of photon orbital angular momentum in the low-frequency radio domain. Phys. Rev. Lett..

[41] Mehmood MQ (2016). Visible-frequency metasurface for structuring and spatially multiplexing optical vortices. Adv. Mater..

[42] Ling XH (2017). Recent advances in the spin Hall effect of light. Rep. Prog. Phys..

[43] Xu HX, Liu H, Ling XH, Sun YM, Yuan F (2017). Broadband vortex beam generation using multimode Pancharatnam–Berry metasurface. IEEE Trans. Antennas Propag..

[44] Yang YJ, Thirunavukkarasu G, Babiker M, Yuan J (2017). Orbital-angular-momentum mode selection by rotationally symmetric superposition of chiral states with application to electron vortex beams. Phys. Rev. Lett..

[45] Liu S, Cui TJ, Zhang L, Xu Q, Wang Q (2016). Convolution operations on coding metasurface to reach flexible and continuous controls of terahertz beams. Adv. Sci..

[46] Karimi E, Schulz SA, De Leon I, Qassim H, Upham J (2014). Generating optical orbital angular momentum at visible wavelengths using a plasmonic metasurface. Light Sci. Appl..

