# Supplementary material for: Interference-assisted kaleidoscopic meta-plexer for arbitrary spin-wavefront manipulation
Source: Light Sci Appl. 2019 Jan 9;8:3. doi: 10.1038/s41377-018-0113-y (PMC6325065; doi:10.1038/s41377-018-0113-y)
Supplement: Supplementary file 1 — Interference-Assisted Kaleidoscopic Meta-plexer for Arbitrary Spin-Wavefront Manipulation [file 41377_2018_113_MOESM1_ESM.doc]

Supplementary Materials

Interference-Assisted Kaleidoscopic Meta-plexer for Arbitrary Spin-Wavefront Manipulation

He-Xiu Xu^1,2,6*†^, Guangwei Hu^1†^, Ying Li^1†^, Lei Han^1,3^, Jianlin Zhao^3^, Yunming Sun^4^, Fang Yuan^2^, Guang-Ming Wang^2^, Zhi Hao Jiang^5^, Xiaohui Ling^6^, Tie Jun Cui^5^, Cheng-Wei Qiu^1*^

^1^Department of Electrical and Computer Engineering, National University of Singapore, Singapore 117583 (Singapore)

^2^Air and Missile Defense College, Air force Engineering University, Xi'an 710051 (China)

^3^School of Natural and Applied Sciences, Northwestern Polytechnical University, Xi'an 710072 (China)

^4^Advanced Technique Department, Key Lab of Aeronautics Computing Technique, Xi'an 710075(China)

^5^State Key Laboratory of Millimeter Waves, Southeast University, Nanjing 210096 (China)

^6^College of Physics and Electronic Engineering, Hengyang Normal University, Hengyang 421002 (China)

^†^These authors contribute equally to this work

^*^Corresponding author: He-Xiu Xu, E-mail: hxxuellen@gmail.com; Tie Jun Cui, E-mail: [tjcui@seu.edu.cn](mailto:tjcui@seu.edu.cn); Cheng-Wei Qiu, E-mail: [eleqc@nus.edu.sg](mailto:eleqc@nus.edu.sg)

**1. Jones matrix for composite meta-atom**

**2. Additional results for meta-atom with asymmetric CP reflections**

**3. Additional results for multiplexed Bessel beam and RCS reduction**

**4. Additional results for multiplexed vortices with versatile beams**

**5. Extension of our strategy to transmission scheme and high frequencies**

**6. Microwave experiments**

1. **Jones matrix for composite meta-atom**

In this subsection, we give detailed information on how to obtain the Jones matrix of the composite meta-atom from those of individual SRR_1_ and SRR_2_. In a general case, the LP wave exhibiting arbitrary polarized angle *θ* is a combination of spin-up and spin-down waves, i.e., $E_{i}=E_{+}^{inc}|+\rangle+E_{-}^{inc}|-\rangle=\left[\begin{matrix} E_{+}^{inc} \\ E_{-}^{inc} \end{matrix}\right]$ with $E_{+}^{inc}=(\cos\theta-i\sin\theta)/2$ and $E_{-}^{inc}=(\cos\theta+i\sin\theta)/2$. Since there are both geometric and orientation differences between SRR_1_ and SRR_2_, the reflective fields of SRR_1_ and SRR_2_ at spin-down state are formulated as

(1)

In a similar manner, the reflective beam under normal illumination of a plane spin-up wave is immediately obtained as

(2)

Then, the reflections of proposed composite meta-atom with negligible mutual coupling between SRR_1_ and SRR_2_ at LP state with any *θ* can be synthesized as

(3)

For a system exhibiting negligible CP cross-polarization component and illuminated by a normally incident CP or elliptically-polarized wave of opposite helicity, Eq. (3) can be simplified as

(4)

1. **Additional results for meta-atom with asymmetric CP reflections**

Here, we give more information about the meta-atom with diode-like asymmetric reflections studied in Fig. 2 in the main text. As can be seen from Fig. S1(a), several reflection zeros are induced by the symmetric and asymmetric modes of SRR_1_ and SRR_2_, accounting for the broadband low cross-polarization reflections of the composite meta-atom by combining a chain of these resonant modes. As expected in Fig. S1(b), the $\text{|}r_{--}\text{|}$ can be continuously modulated by controlling the twist angle of *ψ* within -180^o^~0^o^, coinciding well with the conclusion predicted by theory. The same conclusion can be drawn for $\text{|}r_{++}\text{|}$ by engineering *ψ* within 0^o^~180^o^.

In the main text, we attribute the minor and trivial difference between the FDTD simulations and the theory based on interference analysis to the weak coupling of two SRRs [1]. To further confirm the argument, coupled mode analysis using coupled harmonic oscillator model is exploited where two SRRs are considered as resonators with the coupling strength denoted as g. The FDTD simulated reflection coefficient $\text{|}r_{--}\text{|}$ of Fig. S1(c) can be fitted using following formula [2].

$|r_{--}\text{|}=A\omega^{4}|\frac{(\omega^{2}-\omega_{1}^{2}+i\omega\Gamma_{1})}{(\omega^{2}-\omega_{1}^{2}+i\omega\Gamma_{1})(\omega^{2}-\omega_{2}^{2}+i\omega\Gamma_{2})-\omega^{2}g^{2}}|^{2}$ (5)

where and $\omega_{2}$ are the resonance frequencies; $\Gamma_{1}$ and $\Gamma_{2}$ are the damping coefficients of the individual oscillator. The fitting result shown in Fig. S1(c) indicates that the two resonant frequencies are 8.2 and 9.6 GHz, which agree with the simulation results shown in Fig. S1(a). The obtained value of damping result indicates that $2g<\Gamma_{1}+\Gamma_{2}$, which directly verifies the weak coupling between the two oscillators. Thus, combining the weak coupling and the result in Fig. 2 (main context), it can be concluded that, although the analytical interference result without the analysis of coupling is slightly different from the simulated one, the straightforward and simple model by the destructive interference in the main context could provide an intuitive and nontrivial approach to guide the design of the giant circular dichroism.

From Fig. S1(d)-S1(e), it can be learned that $r_{++}$ manifests almost constant amplitude and ideal reflection PB phase twice the *Φ*. Such orientation-immune high-rate reflections and dispersionless PB phase are crucial for high-efficiency wavefront control. Nevertheless, the ideal PB phase does not hold for $r_{--}$ near the perfect absorption region due to the strong localized fields induced by mutual interference (coupling) between SRR_1_ and SRR_2_ at spin-up state, see the inset of Fig. S1(d). Such coupling has been reduced by several manitudes of factor at spin-down state, giving rise to the unaffected PB phase. Similar constant reflections immune to *Φ* also hold for $r_{+-}$ and $r_{-+}$, see Fig. S1(f).


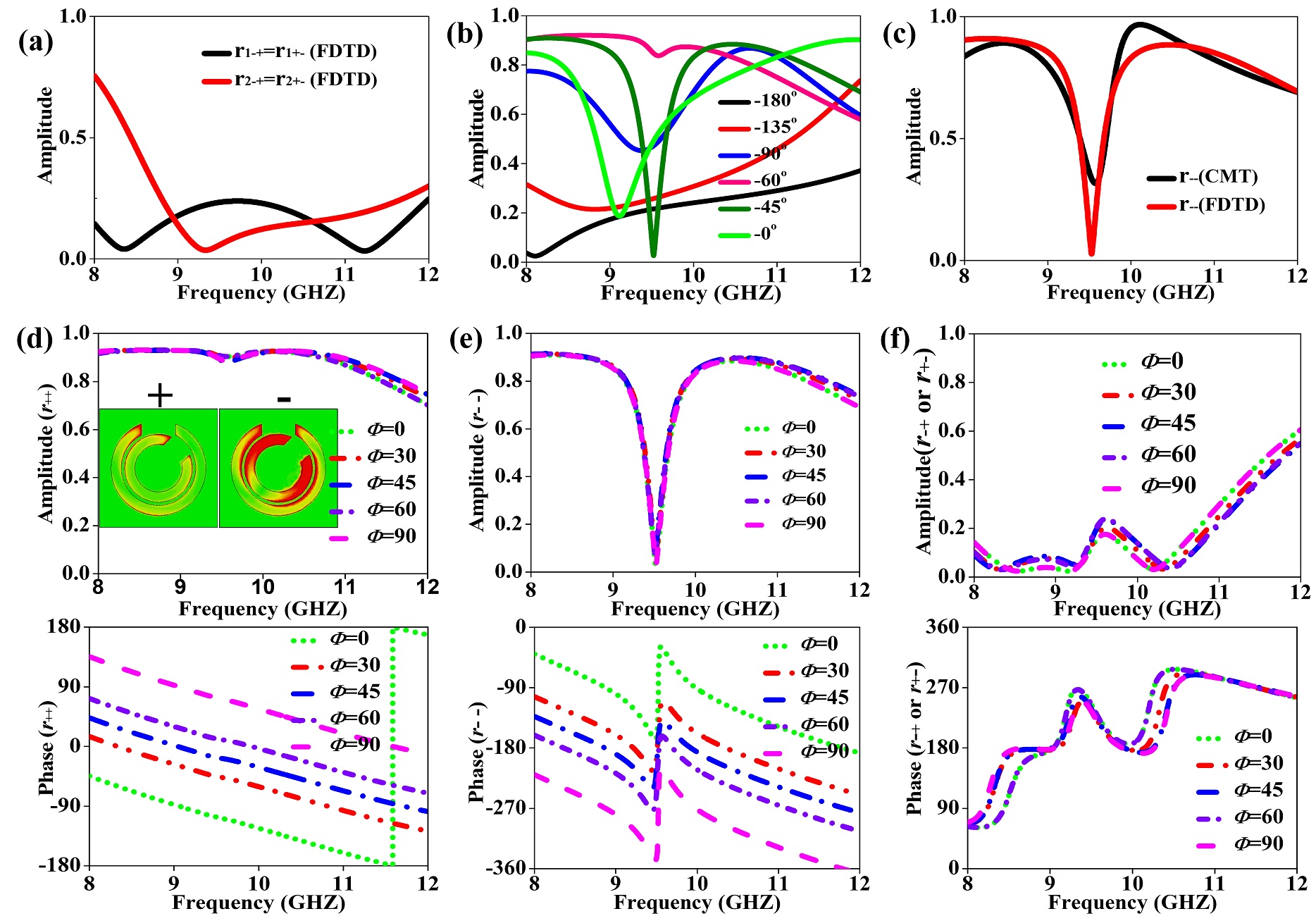


**Figure S1. Characterization of the meta-atom shown in Fig. 2 in the main text.** (a) Broadband cross-polarization reflections of individual SRR_1_ and SRR_2_. (b) Variation of |*r*--| as a function of twist angle *ψ*. (c) Comparison of |*r*--|between FDTD simulation and fitting based on coupled mode theory (CMT). In CMT fitting, we firstly obtain the and $\omega_{2}$, and $\Gamma_{1}$ and $\Gamma_{2}$, the resonance features of each individual split ring resonators from Fig. S1a and then fit the simulation data of whole structures with those fixed parameters to obtain the coupling strength *g*. FDTD simulated reflection amplitude (top panel) and phase (bottom panel) spectra of (d) *r*_++_, (e) *r*-- and (f) *r*_+-_=*r*_-+_ as a function of rotational angle Φ. The inset to Fig. S1(d) shows the near-field *E*_z_ distributions at 9.52 GHz at both spin-down (left panel) and spin-up (right panel) state.

1. **Additional results for multiplexed Bessel beam and RCS reduction**

In this subsection, we afford additional results for the first kaleidoscopic meta-plexer (Fig. 4). As shown in Fig. S2(a), the geometrical parameters are constant while the orientations are spatially changed point by point. As shown in Fig.S2(b) and S2(c), similar distinct functionalities are obtained as those shown in Fig. 4. Combining these results with those shown in the main text at the five typical frequencies, we learn that the propagation beam with localized fields slightly shifts toward larger distances along z axis as frequency increases. This is because the electrically shortened aperture at low frequencies makes our meta-plexer less efficient for long-focal-length imaging. From Fig. S2(d), we again observe an excellent consistency of results between simulations and measurements. The less distorted beam across wide frequencies indicates the generation of non-diffracting Bessel beam of broad operation band.


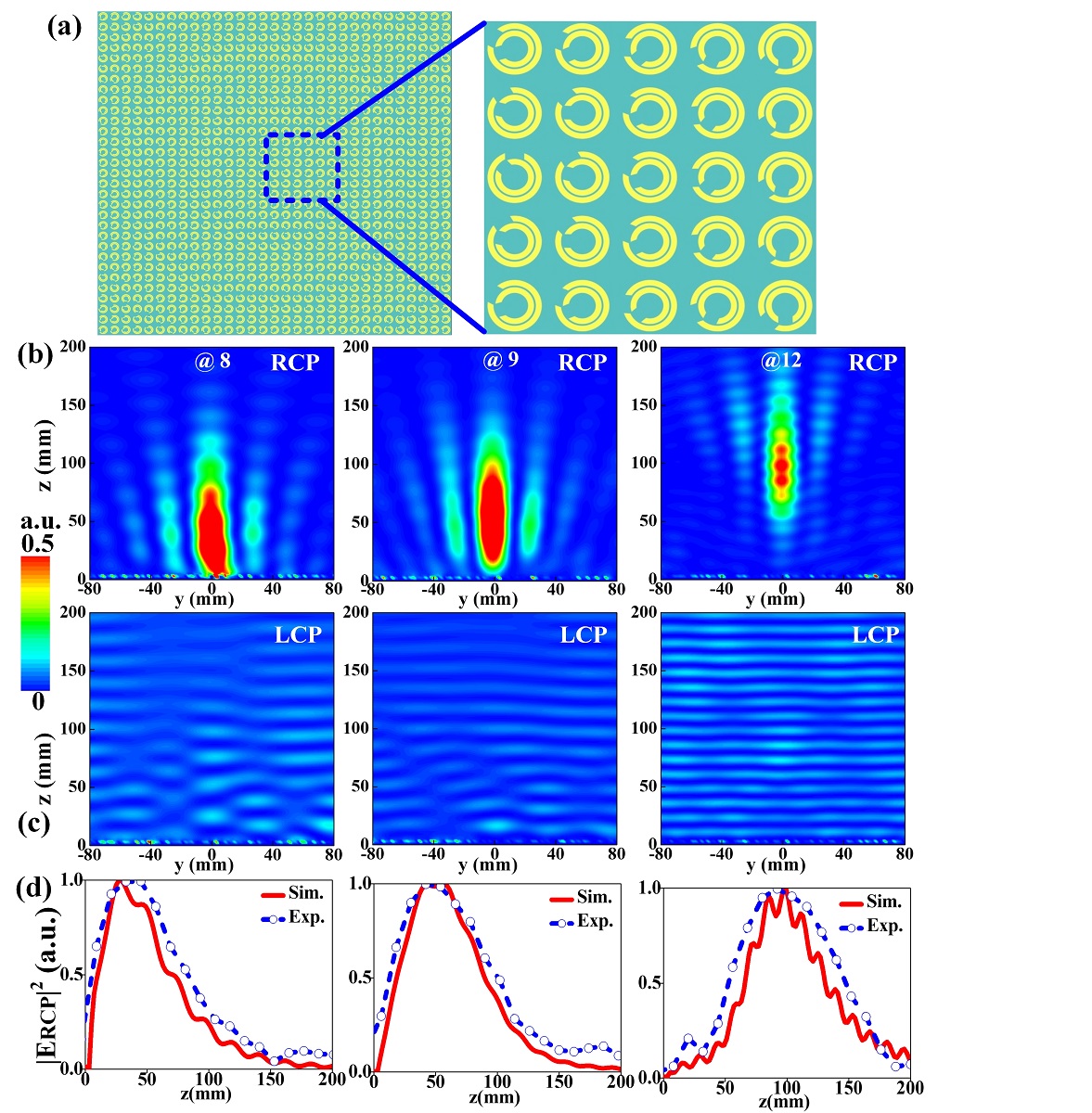


**Figure S2. Characterization of the kaleidoscopic meta-plexer shown in Fig. 4 in the main text.** (a) Perspective and zoom-in-view of the meta-plexer layout. FDTD simulated E-field intensity distributions at 8, 9 and 12 GHz under plane (b) spin-down and (c) spin-up wave illumination. (d) Near-field *E*_RCP_ intensity at spin-down state along the propagating direction (z axis) at x=0 mm at 8, 9 and 12 GHz.

1. **Additional results for multiplexed vortices with versatile beams**

In this subsection, we afford additional results for the second kaleidoscopic meta-plexer shown in Fig. 5. Fig. S3(a) shows the meta-plexer and the pixel-level layout mapped out by the composite phase profile shown in Fig.5. Again, different spatial orientations are observed across the entire metasurface aperture. As plotted in Fig. S3(b) and S3(c), two beams each with a characteristic null at the center are clearly observed at other off-interference frequencies of 8, 9 and 12 GHz, indicating wide operation bandwidth. At *f*_0_=10 GHz, the four beams are pointed at (*θ*; )=(49.3^o^; 45^o^, 135^o^, 225^o^, 315^o^) with *θ* decreasing slightly with frequency. As *f* deviated from *f*_0_, the normal scattering raises slightly as the required phase profile is frequency dependent. As shown in Fig. S3(d), the fingerprint line-spiral wavefronts with severe distortions at the center (singularity) are clearly formed in four regions. As expected in Fig. S3(e), the one-arm spiral phase front is clearly seen on cross section perpendicular to one Bessel beam, coinciding well with the conclusion drawn in the main text.


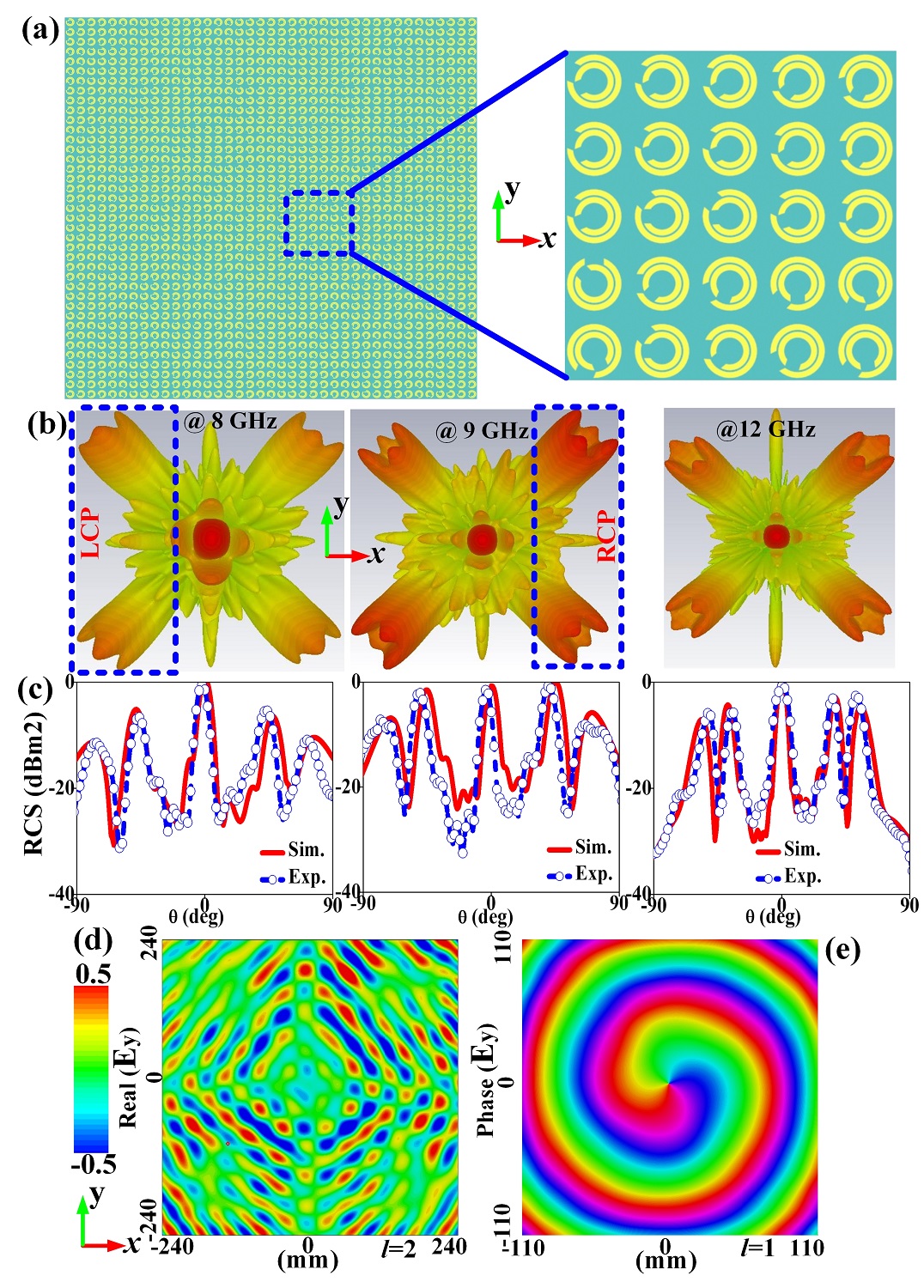


**Figure S3. Characterization of the kaleidoscopic meta-plexer shown in Fig. 5 in the main text.** (a) Perspective and zoom-in-view of the meta-plexer layout. Normalized FDTD simulated and measured (b) 3D scattering patterns and (c) 2D patterns along =45^o^ at 8, 9 and 12 GHz. FDTD simulated near-field (e) Real(*E*_y_) distribution of *l*=2 at z=250 mm (8.75λ) above the meta-plexers and (f) Phase(*E*_y_) distribution of *l*=1 on cross section perpendicular to one Bessel beam at 10.5 GHz.

1. **Extension of our strategy to transmission scheme and high frequencies**

Our proposed strategy and concept even can be directly extended to the transmission scheme and high-frequency region. To support this claim, additional designs and numerical FDTD characterizations are performed and given in Fig. S4 and Fig. S5. As expected in Fig. S4(b), the diode-like asymmetric-spin transmission at spin-up and spin-down state is clearly observed for |*t*-_+_| (0.2) and |*t*_+-_| (near zero) at 9.5 GHz for the transmissive composite meta-atom shown in Fig. S4(a) without the back metallic ground. The origin of spin-selective transmission is again due to the constructive and destructive interferences occurred between the big SRR_1_ and small SRR_2_. Such a claim is strongly supported from the similar transmissive amplitude while -90^o^ phase difference of *t*_+-_ or *t*-_+_ of the two individual meta-atoms, see Fig. S4(c) and S4(d). Nevertheless, we should note that the efficiency of the single-layer transmissive meta-atom (0.2 in current design) is fundamentally limited below 25% because of symmetry requirements, and thus half of the coupled energy radiates back [3]. Moreover, it is also challenging to obtain the strictly uniform transmission amplitude between SRR_1_ and SRR_2_ like that in reflection scheme due to the narrow-band resonance of the SRR. Most importantly, above results also demonstrate that the two SRRs with certain criterion rather than the bottom metal plays a critical role for interference-assisted design rule.


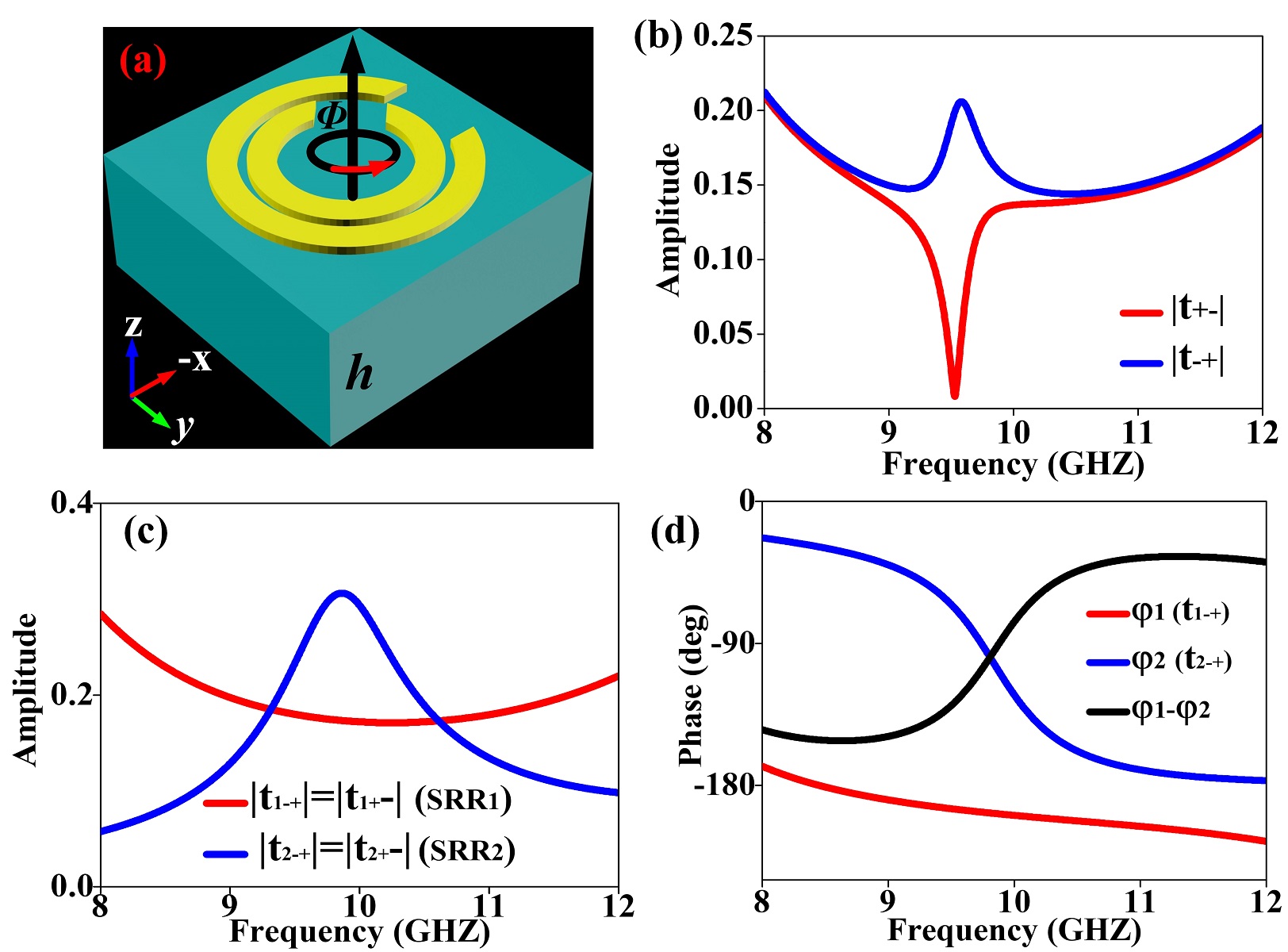


**Figure S4.** FDTD characterization of the transmissive meta-atom by directly removing the back metallic ground from that shown in Fig. 2 in the main text and keeping all parameters constant. (a) Illustration of the topology. (b) Calculated transmissive |*t*-_+_| and |*t*_+-_| of the meta-atom under normally incident spin-up and spin-down plane waves. Calculated (c) amplitude and (d) phase spectra of the individual SRR_1_ and SRR_2_ meta-atom.

As can be seen from Fig. S5, similar spin-selective diode-like behavior is clearly observed for the reflective Au-Si-Au meta-atom designed at near-infrared frequencies, where the reflection of |*r*_++_| and |*r*--| approaches 0.7 and 0, respectively at 176 THz for spin-up and spin-down state. Except for the smaller reflections induced by the larger metallic loss at high frequencies, the quasi-uniform reflection amplitude while -90^o^ phase deviation of *r*_++_ or *r*-- are also expected for the individual SRR_1_ and SRR_2_ meta-atom due to the preserved weak coupling, coincide well with the conclusions drawn in the main text at microwave frequencies. To sum up, the extension of our strategy to the transmission scheme and high frequencies is fully approved from the results shown in Fig. S4 and Fig. S5, indicating the high effectiveness and good generality of our interference-assisted design approach and rule.

**
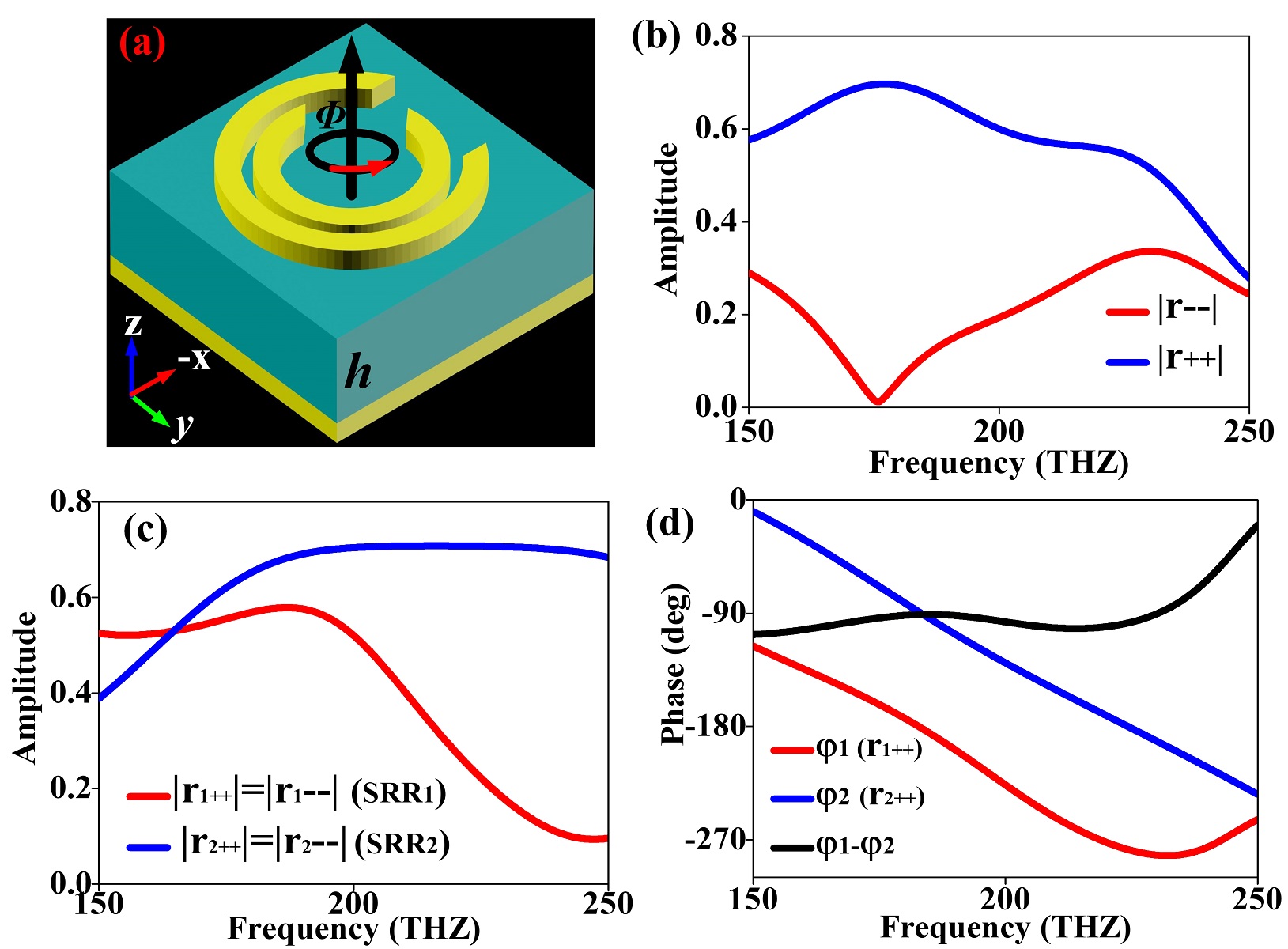
**

**Figure S5.** FDTD characterization of the meta-atom at near-infrared frequencies by scaling the meta-atom shown in Fig. 2 in the main text. (a) Calculated reflective |*r*_++_| and |*r*--| of the meta-atom under normally incident spin-up and spin-down plane waves. Calculated (c) amplitude and (d) phase spectra of the individual SRR_1_ and SRR_2_ meta-atom. The geometrical parameters are detailed as *R*_1_=160, *R*_2_=115, *w*=30, *p*_x_=*p*_y_=400 (unit: nm), *ψ*=45^o^, and the gap width of the inner and outer ring is 120 and 160 mm, respectively. The metal is Au and the thickness of it for both bottom ground and top pattern is 30 nm. The substrate is glass with a dielectric constant *ε*_r_=4.2 and a thickness of *h*=130 nm.

1. **Microwave experiments**


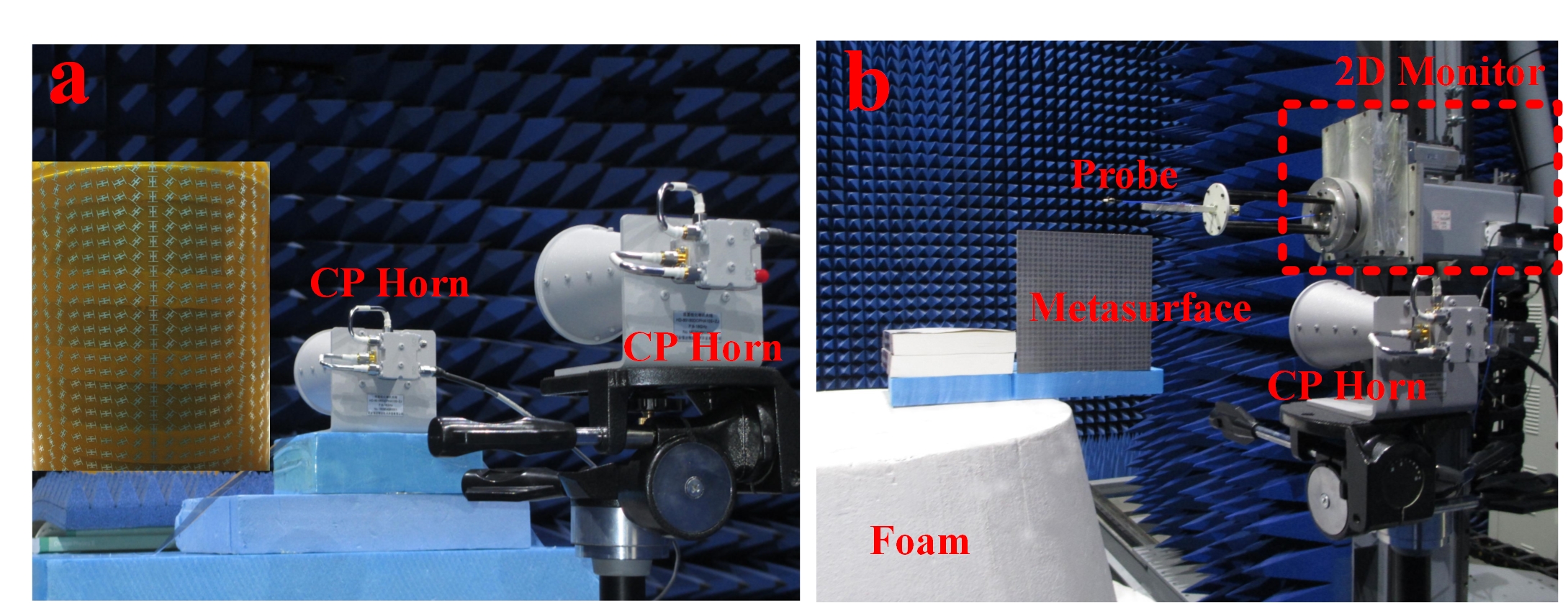


**Figure S6.** Illustration of the (a) far-field and (b) near-field measurement setup in a microwave anechoic chamber.

**References**

[1] Liu N, Liu H, Zhu S, Giessen H. Stereometamaterials. Nat. Photon. 2009; **3**: 157-162.

[2] Wu X, Gray SK, Pelton M. Quantum-dot-induced transparency in a nanoscale plasmonic resonator. *Opt. Express* 2010; **18**: 23633-23645.

[3] Pfeiffer C, Grbic A. Metamaterial huygens’ surfaces: tailoring wave fronts with reflectionless sheets. *Phys. Rev. Lett.* 2013; **110**: 197401.
